# Tango and physiotherapy interventions in Parkinson’s disease: a pilot study on efficacy outcomes on motor and cognitive skills

**DOI:** 10.1038/s41598-024-62786-6

**Published:** 2024-05-24

**Authors:** Giuseppe Rabini, Claudia Meli, Giulia Prodomi, Chiara Speranza, Federica Anzini, Giulia Funghi, Enrica Pierotti, Francesca Saviola, Giorgio Giulio Fumagalli, Raffaella Di Giacopo, Maria Chiara Malaguti, Jorge Jovicich, Alessandra Dodich, Costanza Papagno, Luca Turella

**Affiliations:** 1 Center for Mind/Brain Sciences-CIMeC, University of Trento, Corso Bettini 31, 38068 Rovereto, TN Italy; 2 Department of Medical and Surgical Specialties, Radiological Sciences and Public Health, University of Brescia, Brescia, Italy; 3 Neurology Unit, Rovereto Hospital, Azienda Provinciale per i Servizi Sanitari (APSS) di Trento, Trento, Italy

**Keywords:** Parkinson's disease, Parkinson's disease, Quality of life, Cognitive ageing

## Abstract

Pharmacological treatments in Parkinson’s disease (PD), albeit effective in alleviating many motor symptoms, have limited effects in non-motor signatures as cognitive impairment, as well as in other aspects included postural instability. Consequently, complementary interventions are nowadays a prerogative of clinical practice managing PD symptomatology. In this pilot longitudinal study, we recruited twenty-four PD patients participating in one of two interventions: adapted Argentine Tango or group-based physiotherapy. Participants underwent a motor and neuropsychological evaluation before and after four months of activities, carried out twice a week. We found a general stabilization of motor and cognitive abilities, with significant improvements in several motor skills, mainly pertaining to static and dynamic balance, similarly in both groups. At cognitive level, we measured a significant improvement in both groups in the Action Naming task. Interestingly, only PD patients in the Tango group improved their performance in the test measuring facial emotion recognition. These findings highlight the crucial role that physical activities have in the stabilization and slowdown of disease’s progression in PD. They further highlight the beneficial effects of a group-based physical intervention, which, especially in the case of Tango, could lead to behavioral ameliorations in domains other than the motor, such as emotion recognition.

## Introduction

Movement disorders are within the cardinal features of Parkinsons’s Disease (PD), although non-motor features, such as insomnia, hyposmia, cognitive decline and depression, characterize the clinical phenotype^1–3^. People with PD suffer from motor disabilities which highly compromise several aspects of daily life. While resting tremor has a bearing on fine-grained movements, rigidity, bradykinesia and postural instability impact on the patient’s capability of being autonomous and self-sufficient, especially in the advanced stages of the disease^4–7^. Similarly, non-motor symptoms have a profound impact on ordinary tasks of everyday life of PD people, given that they include sleep disorders, perceptive (visual, olfactory) alterations and cognitive decline, among others. As a results, perceived quality of life in PD people is dramatically affected, with a possible exacerbation of mood disorders^8^. Clinical interventions thus aim at slowing the pace of motor and non-motor symptoms decline, trying to manage the whole spectrum of PD symptomatology. To date, pharmacological intervention with levodopa (L-dopa) is the most efficacious approach in contrasting motor-sign severity in PD^9–11^. L-dopa drug directly acts on the degeneration of dopaminergic neurons in substantia nigra pars compacta, by suppling the dopamine depletion in the basal ganglia-thalamus-cortical circuit^12–15^. Notwithstanding, chronic levodopa treatment could itself lead to pathological complications related to aversive motor effects, such as L-dopa-induced dyskinesias and motor response oscillations^16–18^. Furthermore, L-dopa has limited if any effects on several PD symptoms such as cognitive impairment, speech disturbance, postural instability and freezing of gait^19^. Recent evidence, accordingly to the dopamine overdose hypothesis^20^, suggests also that dopaminergic therapy has a detrimental effect on inhibitory control in the first stages of the disease, turning to beneficial in later stages^21^.

These observations converge for identifying complementary interventions in the management of PD pathology and progression, as a pivotal support of the pharmacological medication treatment. Among those complementary interventions, physical activity represents one of the main protective factors against ageing and neurodegenerative processes at large^22–25^ and it is nowadays considered one of the most effective agents in promoting symptoms alleviation in PD^26–29^. Physical activity in PD has been shown to foster improvements particularly in motor skills: mobility, gait and balance seem to improve after physical exercises^27,30–32^. Notably, beneficial effects of physical activity are not confined to motor abilities, but seem to extend to non-motor features and different domains of cognition, especially executive functions^33,34^. Accordingly, it is known that regular physical activity promotes neural plasticity in PD^35^, by inducing increased corticomotor excitation, variation in grey matter volume and changes in brain derived neurotrophic factor levels^36^.

Within physical activity, in its broader meaning^37^, physiotherapy is one of the most applied methods of neurorehabilitation in PD^38–42^. Physiotherapy entails programmed and specific exercises to be performed under the supervision of licensed physiotherapists with previous experience in neurodegenerative/neurological diseases. The proposed exercises are specific for the main impairments of PD—including gait, mobility, posture and balance—providing significant improvements^38,43^.

In the last decades, dance has also gained considerable attention as a complementary treatment for PD. The manifold nature of dance interventions—which build upon memory processes, rhythmic abilities, emotional and social elements—has the potential to affect more than the motor domain. Accordingly, it has been shown that different forms of dance positively impact on motor, non-motor symptoms and quality of life in PD^44–52^.

Among the plethora of dance typologies, tango—adapted for PD—is widely used in PD management^53,54^. Aside from being a multifaceted activity, as other kinds of dance, tango requires to perform movements specifically involving several of the main motor domains impaired in PD: from static and dynamic balance to postural instability and gait. Furthermore, during tango classes, participants assume both the leading and the following roles. This element is particularly important because it allows participants to move both forward and backward by implementing both intentional (self-programmed) movements and externally directed movements. Importantly, tango dancing incorporates an embrace, fostering a sense of “protected guided movements” for the PD subject dancing with a healthy control, regardless of the dancing role.

Not surprisingly, tango has been shown to improve both motor and non-motor symptoms of PD^55–63^. Recent evidence also suggests that tango has a positive impact on mood and it provides a general reduction of symptoms, promoting social engagement and facilitating person-to-person interactions between patients and healthy volunteers^44^. Despite the positive evidence of the efficacy of tango on PD symptoms, its precise effect on motor and non-motor features of PD is still unclear. This might be due to the high degree of variability in: (1) the implementation of the interventions across studies and (2) the adopted control group to which the effect of tango is compared to^54,64^. Given this puzzling picture, further effort establishing the effects of different kinds of complementary interventions in the management of PD is needed.

To this end, the present study aims to implement an intervention protocol for PD as a complement to pharmacological therapy, comparing two group activities: group-physiotherapy (PG) and adapted Argentine Tango (TG). While there are studies comparing tango and various forms of physical exercises in PD^55^, a structured and sustained program assessing the validity of group-therapy in the forms of physiotherapy or Argentine Tango, is still lacking. Two distinct groups of PD patients participated in a 4-month group intervention based either on physiotherapy exercises or Argentine tango dance. We assessed the clinical profiles of the patients to obtain a comprehensive evaluation of the protocol effects on cognition and behaviour by means of tests in the domain of motor abilities, cognitive skills and mood. Furthermore, we explored the variability of the cognitive and behavioural changes after these physical activities at the individual level, by computing the true inter-individual difference in response to the intervention^65^, with the aim to provide indications for future adoption of these two protocols in the clinical setting.

## Results

### Adherence and group comparison at baseline

Twenty-two participants completed the assessment phases and at least 60% of the intervention sessions, with the exception of one participant who did not perform the motor assessment in the POST phase. Reasons for excluding participants from the final sample were: (1) not having completed the post-intervention assessment and (2) having a percentage of participation at the intervention lower than 60%. Dropouts were not related to the intervention in one case (injuries occurred during the intervention period and other/competing commitments), but not in the other one (the patient was not inclined to follow the intervention we proposed after randomization).

Average percentage of attendance was 92.71 ± 7.69 for the TG (n = 12) and 80.94 ± 13.51 for PG (n = 10). The final sample for the analyses was constituted by 22 participants for neurocognitive measures, and 21 participants for motor measures.

At baseline, there were no significant differences between the two groups for demographic features (all *p* > 0.10, see Table 1), motor sign severity (UPDRS-III, *p* = 0.48, see Table 1), all the motor measures (all *p* > 0.06, see Table S3), all the cognitive measures (all *p* > 0.12, see Table S4), all the subscales of the PDQ-39 (all *p* > 0.06, see Table S1) and all the affective measures (all *p* > 0.21, see Table S1). The only exception we found, was related to the total score of the Parkinson Anxiety scale, for which we had a significant difference at baseline between groups (t_(21)_ = 2.23, *p* = 0.04, Cohen’s d = 0.91, Table S5).

**Table 1:** Clinical features of the experimental sample.

	TG (n = 12)	PG (n = 12)	Statistics
Sex	5F, 7 M	5F, 7 M	U = 72.000, * p* = 1.000, r = 0.000
Age in years (range)	68 ± 8 (51–79)	70 ± 7 (58–82)	t = − 0.595, * p* = 0.558, Cohen’s d = − 0.243
Education in years (range)	14 ± 4 (8–19)	11 ± 4 (6–18)	t = 1.685, * p* = 0.106, Cohen’s d = 0.688
Disease duration in months (range)	59.73 ± 18.42 (24–84)	83.17 ± 66.42 (14–252)	U = 57.500, * p* = 0.617, r = − 0.129
UPDRS-III (range)	20.27 ± 11.14 (3–34)	17.00 ± 10.75 (3–34)	t = 0.717, * p* = 0.481, Cohen’s d = 0.299
Hoehn & Yahr (range)	1.91 ± 0.70 (1–3)	1.83 ± 1.11 (0–4)	U = 71.000, * p* = 0.769, r = 0.076
LEDD (mg)	459.00 ± 178.78	49.400 ± 281.25	t = 0.318, * p* = 0.754, Cohen’s d = 0.136

TG, Tango group; PG, Physiotherapy group; UPDRS-III. Unified Parkinson’s Disease Scale—part III.

### Motor sign severity, affective measures and quality of life

For UPDRS-III, we found a significant main effect of the factor *Session* (F_(1,19)_ = 9.15, *p* = 0.007, η^2^ = 0.03, BF_10_ = 6.87) indicating an improvement (decreased values) from PRE (mean = 19.62; sd = 10.64) to POST (mean = 16.24, sd = 8.31) session. We did not find any significant effect of interaction between *Group* and *Session* (F_(1,19)_ = 0.34, *p* = 0.56, η^2^ = 0.001), thus showing that the improvement on motor sign severity was not significantly different between the two groups. S1.

Results on affective measures indicates no significant difference between PRE and POST sessions in the two groups regarding levels of anxiety (Parkinson Anxiety Scale, all *p* > 0.07), depression (Geriatric Depression Scale, all *p* > 0.09) and apathy (Apathy Evaluation Scale, all *p* > 0.3).

**Figure 1:**
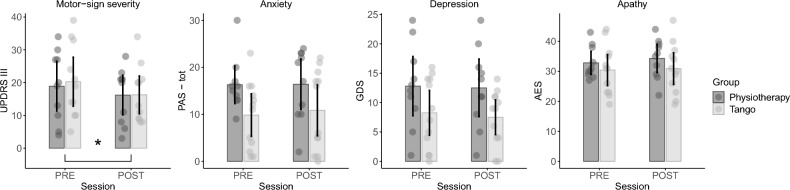
Motor-sign severity and affective measures. Comparison between pre and post evaluation sessions are shown in different plots, pertaining to motor-sign severity, anxiety, depression and apathy. Dark grey represents the physiotherapy group (PG), light grey represents the tango group (TG). UPDRS-III = Unified Parkinson’s Disease Rating Scale, part III; PAS-tot = Parkinson Anxiety Scale, total sub-score; GDS = Geriatric Depression Scale; AES = Apathy Evaluation Scale. * indicates a significant main effect of Session (*p* < 0.05).

The questionnaire on Perceived Quality of Life (PDQ-39) showed only a significant effect of *Session* for the subscale related to Mobility (F_(1,19)_ = 4.87, *p* = 0.04, η^2^ = 0.009, BF_10_ = 1.67), whereas all the other effects for all the other subscales (Total score, Activity of Daily life, Emotional Wellbeing, Stigma, Social Support, Cognition, Communication and Bodily Discomfort), were not statistically significant (all *p* > 0.1, see Fig. 1).

### Motor skills assessment

#### Static balance

The mini-BESTest showed a significant main effect of *Session* (F_(1,20)_ = 24.11, *p* < 0.001, η^2^ = 0.05, BF_10_ = 54.12), a significant effect of interaction *Group* x *Session* (F_(1,20)_ = 6.25, *p* = 0.021, η^2^ = 0.01, BF_10_ = 92.53), but not significant results for the factor *Group* (F_(1,20)_ = 0.14, *p* = 0.72, η^2^ = 0.006). Mini-BESTest scores improved (increased) from PRE to POST session (PRE: mean = 25.27, sd = 6.52; POST: mean = 27.86, sd = 5.57). Post-hoc analysis revealed that the interaction effect was driven by a significant difference between PRE e POST session in the Physiotherapy group (t_(9)_ = 7.50, *p* < 0.001, Cohen’s d = 2.37). For the Berg Balance Scale, there was a significant effect of *Session* (F_(1,20)_ = 6.09, *p* = 0.023, η^2^ = 0.04, BF_10_ = 1.98) and not significant effects neither for *Group* (F_(1,20)_ = 0.013, *p* = 0.91, η^2^ < 0.01), nor for the interaction term (F_(1,20)_ = 1.72, *p* = 0.20, η^2^ = 0.01). Scores on the Berg Balance Scale improved (increased) from PRE (mean = 51.50, sd = 5.57) to POST (mean = 53.36, sd = 4.50) session.

#### Dynamic balance

For the Four Square Step, considering the mean values (FSS-mean), results indicated a significant effect of *Session* (F_(1,20)_ = 11.06, *p* = 0.003, η^2^ < 0.09, BF_10_ = 11.47), with the main effect of *Group* (F_(1,20)_ = 0.01, *p* = 0.92, η^2^ < 0.01), and the interaction effect (F_(1,20)_ = 0.55, *p* = 0.47, η^2^ = 0.005), not reaching significant levels. Scores on the Four Step Square improved (decreased) from PRE (mean = 11.09, sd = 2.12) to POST (mean = 9.70, sd = 2.44) phase.

Results on the TUG test revealed a significant effect of the factor *Session* (F_(1,20)_ = 20.17, *p* < 0.001, η^2^ = 0.15, BF_10_ = 76.24). There were no significant effects for *Group* (F_(1,20)_ = 0.74, *p* = 0.40, η^2^ = 0.024) and for the interaction term (F_(1,20)_ = 2.33, *p* = 0.14, η^2^ = 0.02). Values on the TUG decreased from PRE (mean = 9.50, sd = 2.37) to POST (mean = 7.58, sd = 2.39), indicating performance improvement.

For the Ten meters walking test – Fast condition (10mFast), considering the mean values, we did not find any significant main effect or interaction (all *p* > 0.2).

#### Lower-Limb mobility and Gait

Results on the six-meter walking test (6mwt) indicates that there was not significant difference between the two groups, in the distance travelled, between PRE and POST session (all *p* > 0.1). When considering the mean values, for both the Five-time seat to stand (5STS) and the Thirty-seconds seat to stand (30STS), we did not find any significant results (all *p* > 0.1).

For the Ten meters walking test – slow condition (10mFast), considering the mean values, we did not find any significant main effect or interaction (all *p* > 0.2).

#### Upper-Limb mobility

The nine-hole peg test (9hpt) indicates a comparable performance between PRE e POST sessions, between the two groups, for both the right and left hand (not significant results at the ANOVAs, all *p* > 0.4).

#### Questionnaires

**Figure 2:**
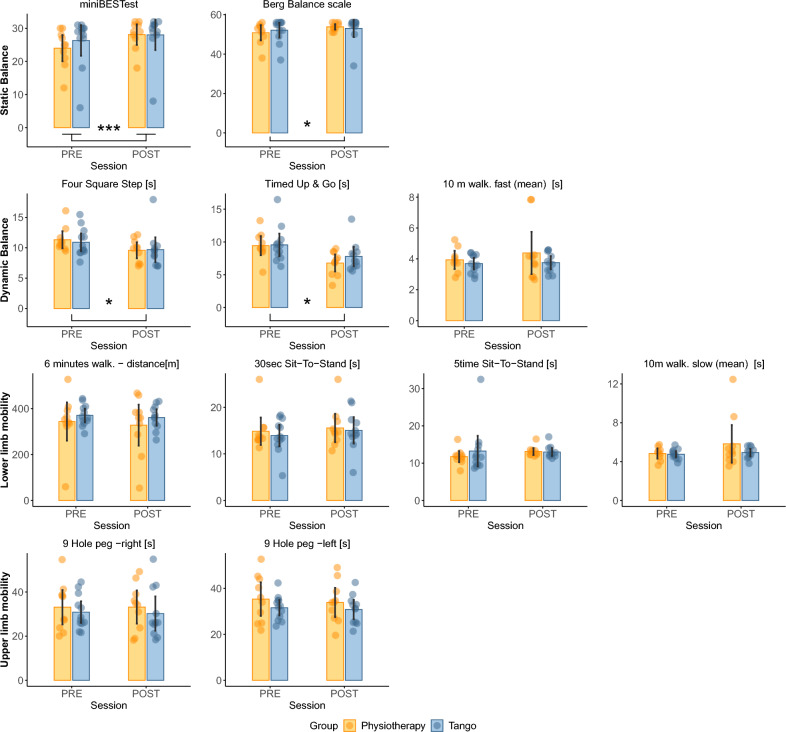
Motor Skills. Comparison between PRE and POST evaluation sessions are shown in different plots, pertaining to motor skills. Different motor domains are presented for each row of the panel: static balance, dynamic balance, lower limb mobility and upper limb mobility, respectively. Yellow represents the physiotherapy group (PG), blue represents the tango group (TG). * indicates a significant main effect of Session (*p* < 0.05); *** indicate a significant Group x Session interaction effect (*p* < 0.05).

We delivered to participants several questionnaires related to fatigue (Fatigue Severity Scale), freezing of gait (Freezing of gait test), balance confidence (Activity-Specific Balance Confidence Scale) and falls (Falls Efficacy Scale International). We did not find any significant change between session and across groups (main effects and interactions in the ANOVAs, all *p* > 0.1). Figure 2 represents all the results related to motor skills assessment.

### Cognitive assessment

The whole neuropsychological assessment comprised a manifold of cognitive measures spanning different domains: memory, attention, executive functions, visuo-spatial processing, language and emotion recognition. Given the limited time between PRE and POST evaluation (around 6 months), we did not have specific hypotheses about possible performance changes in the cognitive domain. However, given the group format of our interventions and the inherently social nature of this approach, a change pertaining to the larger domain of social cognition, such as in emotion recognition, could be expected. Our findings reported a few significant results regarding language and visual emotion recognition.

In the language domain, we reported significant effect of *Session* for Action Naming task (F_(1,20)_ = 8.05, *p* = 0.01, η^2^ = 0.06, BF_10_ = 3.69) and a marginal significant effect of *Session* for the Object Naming task (F_(1,20)_ = 4.04, *p* = 0.058, η^2^ = 0.026). In both cases, main effect of *Group* (Action Naming: F_(1,20)_ = 1.79, *p* = 0.19, η^2^ = 0.06; Object Naming: F_(1,20)_ = 2.63, *p* = 0.12, η^2^ = 0.098) and the interaction effect (Action Naming: F_(1,20)_ = 0.86, *p* = 0.36, η^2^ = 0.007; Object Naming: F_(1,20)_ = 0.06, *p* = 0.81, η^2^ < 0.001) were not statistically significant. Scores at both naming tasks improved (increased) from PRE (Action Naming: mean = 47.34, sd = 4.14; Object Naming: mean = 46.99, sd = 1.48) to POST (Action Naming: mean = 48.98, sd = 2.29; Object Naming: mean = 47.41, sd = 1.13) session.

We found an interaction effect *Group* x *Session* in the Ekman 60 Faces Test (F_(1,20)_ = 5.56, *p* = 0.029, η^2^ = 0.02, BF_10_ = 2.07). Post-hoc analyses (paired t-test) revealed that the interaction effect was resulting from a significant improvement in the Tango group (t_(11)_ = 2.03, *p* = 0.034, Cohen’s d = 0.57; PRE: mean = 49.92, sd = 5.39; POST: mean = 51.67, sd = 4.67) and a decreased level of performance, albeit not significant, in the Physiotherapy group ((t_(9)_ = 1.39, *p* = 0.9, Cohen’s d = 0.44; PRE: mean = 47.27, sd = 6.01; POST: mean = 45.57, sd = 7.35). When looking at the subscale of the test, which refer to single emotions (Surprise, Happiness, Fear, Anger, Disgust and Sadness), we found a significant interaction effect *Group* x *Session* (F_(1,20)_ = 9.06, *p* = 0.007, η^2^ = 0.05) in the scale of sadness. Post-hoc analyses (paired t-test) revealed that the interaction effect was resulting from an opposite direction of the effect in the two groups. We reported and improvement, albeit not significant, in the Tango group (t_(11)_ = 1.87, *p* = 0.087, Cohen’s d = 0.54; PRE: mean = 8.00, sd = 1.65; POST: mean = 8.67, sd = 1.15) and a significant decreased level of performance in the Physiotherapy group ((t_(9)_ = 2.28, *p* = 0.048, Cohen’s d = 0.72; PRE: mean = 7.9, sd = 2.6; POST: mean = 6.8, sd = 2.04).

In all the other measures (attentive Matrices, Facial Recognition Test, Trial Making Test, Stroop test, Phonemic and Semantic Verbal Fluency) there were no significant main effects or interaction effects (all *p* > 0.05).

**Figure 3:**
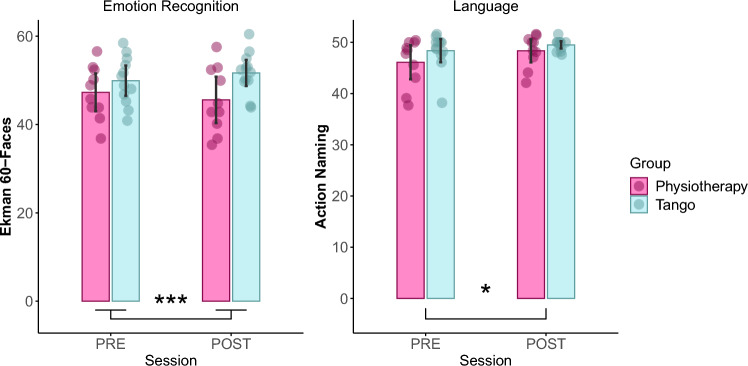
Significant results on cognition. Comparison between PRE and POST evaluation sessions are shown in different plots, pertaining to significant results obtained in the cognitive domain: visual emotion recognition (Ekman 60 Faces test) and language (Action Naming test). Pink represents the physiotherapy group (PG), light blue represents the tango group (TG). * indicates a significant main effect of Session (*p* < 0.05); *** indicate a significant Group x Session interaction effect (*p* < 0.05).

We found a main effect of *Group* in the Judgement of Line Orientation test (F_(1,20)_ = 4.37, *p* = 0.049, η^2^ = 0.15) and the Stroop task, considering the time of completion (F_(1,20)_ = 4.92, *p* = 0.038, η^2^ = 0.16). Significant results on cognitive assessment are presented in Fig. 3.

### True inter-individual differences

Considering the significant results described in the previous section, we did not report any significant true inter-individual difference (SD_R_, see Method section for clarification) for each of the considered variables (UPDRS-III, miniBESTtest, Berg Balance Scale, Four Step Square, Timed Up and Go, Action Naming and Ekman-60, all *p* > 0.1), suggesting no true inter-individual difference in response to both the tango intervention and the physiotherapy intervention. Considering the other variables, we found significant true inter-individual differences (SD_R_) in the physiotherapy group compared to the tango group in the following measures: 6 min walking test – distance (*p* < 0.001), 10 m walking test (*p* < 0.001), TMT A (*p* < 0.001), TMT B (*p* < 0.001), TMT B-A (*p* < 0.001), Stroop test – time (*p* < 0.001) and in the PAS total score (*p* = 0.021). These significant SD_R_ suggest a higher variability in the effect of the training for the physiotherapy, as it showed a higher SDs of change in this group compared to the tango group (see Table S6).

## Discussion

Complementary interventions to pharmacological treatment are nowadays an essential aspect of the management of PD pathological manifestations. In the present study, we compared two intervention protocols for PD based either on group-physiotherapy or Argentine tango dance. We adopted two structured interventions (described in Supplementary Information) in an extensive program lasting four consecutive months (1 h, twice a week, for a total of 32 h). Our results showed an improvement in motor abilities especially regarding static and dynamic balance, in both groups. Preservation of motor skills were reported in all the other measures as well. In the cognitive domain, we reported a general performance stabilisation with a significant improvement regarding language (naming actions) in both groups. Intriguingly, we measured a significant improvement in visual emotion recognition in the tango group, but not in the physiotherapy group. Affective measures of anxiety, depression and apathy did not change after the intervention, while perceived quality of life in relation to general aspects of mobility improved after the protocol in both groups.

In the present investigation, the choice to implement interventions based on group activities of physiotherapy and tango dance was grounded on previous evidence suggesting that both activities have the potential to improve motor and non-motor symptoms of PD^43,50^. Physiotherapy has been and, it is even now, the standard physical therapy for PD^41^. The impact on motor and non-motor features has been linked to the specificity of the proposed exercises (e.g., focusing on balance, muscle strength, resistance, stability, gait and falls). Notably, it is not still clear which specific protocol of physiotherapy could lead to greater benefit in PD (in terms of both improvement or stabilisation of symptoms), given that several techniques (i.e., conventional, treadmill training, exercise, cueing) have been proven to be similarly effective and substantial differences in measures of outcomes prevented a consistent comparison^40^. Accordingly, there is still not a consensus on which approach to adopt as physiotherapy intervention in PD, as different types of physiotherapy impact differently on specific motor outcomes (such as motor sign severity, balance, gait)^43^. Here, we adopted a group-based approach of conventional physiotherapy, providing specific exercises with progressive difficulty and pertaining to four main domains: balance skills, muscle strength, body movements coordination and social engagement promoted by the group context.

Tango, on the other side, has also been shown to be a feasible intervention for PD patients. Although a previous review on the topic emphasized the heterogeneity of the tango programs delivered to PD patients and the lack of consistency in reporting protocol details, it also highlighted the high degree of applicability of such an intervention in PD population^54^. Theoretical improvements of tango therapy have been mainly hypothesised to be linked to the features of music-based movement therapies^66^. Accordingly, it enables external cueing offered by music, as an auditory stimulus facilitating pace and rhythm of movements^56^. Tango dance integrates exercises of balance and mobility by stepping forwards and backwards and by following the movements of the partner. Moreover, it involves cognitive strategies related to attention and memory to perform the right sequences of movements. These former elements have been primarily linked to the potential positive cognitive outcomes of tango and dance in general, which however has been shown to be limited^67^ or highly heterogeneous^64^.

Following our protocol, motor sign severity in PD (UPDRS-III scores) decreased (improved) after the intervention with no significant differences between TG and PG groups. This positive outcome confirms the efficacy of sustained physical activity in promoting a, although limited, relief from motor impairment. The absence of significant differences between groups suggests comparable effects of these kinds of intervention on global motor sign severity in PD. Anecdotally, we reported a mean improvement of 3.38 points in the UPDRS-III scale. Not all the participants improved from PRE to POST session, however, we measured an improvement in 72% of the patients (4 patients in the PG and 2 in the TG did not improve). Similar heterogeneous results on motor sign severity improvement have been already reported^60^. Decreased levels of motor sign severity have been previously reported after tango intervention in comparison with a control group with no intervention^64,68^ or exercise group^64,69^. Considering physiotherapy interventions^43^, improved motor sign severity in PD (UPDRS-III) has been previously reported after conventional physiotherapy^70,71^, Nordic walking^72,73^, balance and gait training^74^. In this framework, our results suggest that group-based interventions based on structured physical activity in general (such as physiotherapy or tango dance) could have the potential to alleviate motor sign severity in PD.

We did not report any significant difference related to affective measures pertaining to anxiety, depression and apathy. Notably, although we measured a marginal significant main effect of group related to the Parkinson Anxiety scale, which indicates an overall higher level of anxiety in the physiotherapy group (Group: F_(1,20)_ = 4.31, *p* = 0.051; TG: mean PRE = 9.83, POST = 10.83; PG: mean PRE = 16.3, POST = 16.4), the difference between PRE and POST session was not significant between and across groups. This observation confirms that both the physiotherapy and tango intervention did not lead to significant changes in the levels of anxiety.

Also previous investigations adopting physiotherapy reported no improvements in the PAS^75^. However, anxiety has not been usually considered among the outcomes of complementary interventions^43,48,54,64^.

Given that symptoms of depression were slightly present in our sample (only 2 out of 22 patients showed a serious level of depression at baseline; 10 other patients with minor levels of depressive symptoms), it is reasonable to suppose that significant changes, whenever would have occurred, were difficult to capture anyway. A similar reasoning could be applied to the results on apathy, for which we did not measure a significant difference from pre to post training. Although a mood improvement (especially from depressive and apathy feelings) is somehow one of the desired outcomes in intervention protocols in PD, an absence of significant changes in those areas has been already shown in previous studies adopting tango^51,69^. Previous evidence on tango are quite scarce^44^, however positive effects on depressive mood, as measured with the Beck Depression Inventory, have been shown using dance protocols^76^ and conventional physiotherapy interventions^77,78^. Notably, similarly to the present study, in the cited studies the number of patients showing severe levels of depression was limited.

Results on mood changes after intervention with physical activity are quite heterogeneous and surely require further investigations. Probably, the scales used to measure these psychological constructs are very different between studies or they are not sensitive enough to capture even small changes occurring in patient’s mood after a few months of activity. Alternatively, the proposed interventions, although promoting social interaction and positive feelings within the group sessions, are not specific and effective enough to determine significant changes in mood.

Interestingly, a few studies adopting interventions based on active theatre have been shown to be effective in improving levels of depression, apathy and event stigma^79,80^. These findings suggest that an alternative approach, requiring creativity, engaging social interactions and simulation of real-life situations, might be particularly suitable as a rehabilitation form for PD patients. Impacting non-motor features such as the psychological well-being of the patients, it could dramatically change the every-day living of the person and her/his caregiver.

Nonetheless, we reported a significant effect of session related to the PDQ-39 mobility subscale, suggesting an improvement of perceived quality of motor mobility in daily life, in a comparable way in both groups. The intervention proposed thus prompted an improvement in subjective feelings of functional mobility. This result is partially in line with the behavioural improvements measured objectively in the motor scales, and it advocates for a substantial repercussion of the physical activities in everyday life of PD patients. Our results replicate previous studies implementing home-based exercise intervention^81^ and tango dance^82^. In this latter study, authors showed an improvement in the mobility, social support and summary index subscales of the PDQ-39 of the adapted tango dance group in comparison to those performing other types of dance.

We found significant performance improvements in static and dynamic balance, whereas a stabilisation of motor skills was evident for lower limb mobility, walking and upper limb mobility. We found an improvement on static balance measured by two specific motor tests, i.e. as the miniBESTest and the Berg Balance Scale. These results replicate previous findings in protocols using conventional physiotherapy or alternative types of physiotherapy^83–86^ and studies using Argentine tango or adapted tango^55,68, 87^. Comparably, we measured a significant improvement in the domain of dynamic balance as measured with the Four Square Step and the Timed Up & Go test, which is often considered an index also of functional mobility. Again, previous research also showed an improvement in those tests with both physiotherapy^70,88–90^ and tango interventions^60,69^.

However, contrasting evidence on physical activity and physiotherapy in PD showed an absence of significant changes in the aforementioned measures, as well as performance improvement in domains for which we only showed a stabilisation of performance (i.e., gait, lower and upper-limb mobility) (see Radder et al.^43^ for a review). Here, we showed that a group-based intervention of physiotherapy induced significant changes in motor performance in aspects critically related to balance, which is one of the core motor impairments in PD.

Likewise, literature on tango is highly inconsistent, with studies showing a heterogeneous improvement in motor performance in the domains of balance, gait and postural stability (see Axelerad et al.^64^, Berti et al.^54^ for reviews). Also, our tango-based intervention had beneficial effects mainly in terms of balance, with stabilisation of performance in the other domains.

We propose that both a highly specific physical activity such as physiotherapy and a less-specific, although more complex and multifaced social activity, such as Argentine tango, could lead to performance improvement in the domain of motor abilities, which is one of the cardinal aspect of PD pathology.

Overall, our findings point to short-term outcomes, while a more enduring effect of complementary intervention in PD would be the longstanding objective. Previous evidence provides promising indications in this direction, since longer protocols of tango dance interventions led to improvements in balance, gait, lower limb and also upper limb mobility^68,87^.

Previous evidence of the effects on cognition of different types of complementary intervention in PD is still puzzling, with some studies showing significant improvements and others not^34,43,44,46,64^. Previous studies demonstrating cognitive improvements adopting tango intervention in PD showed enhanced performance for instance in executive functions^51,91^, a trend towards improvement in global cognition (MoCA) and executive functions^69^, and improvements in spatial cognition^91^. Comparably, investigations adopting physical exercises interventions in PD showed heterogeneous improvements in cognition^34^. For instance, treadmill training has been proved to enhance performance in executive functions and mental flexibility^92^.

Within this disparate constellation of results, we largely measured a stabilisation of cognitive performance in our PD sample. Given the limited period between pre and post evaluation sessions, which was roughly of five to six months, we did not expect large effects in this direction. Nonetheless, we reported interesting improvements in the domains of language and visual emotion recognition.

Our first interesting finding pertains to a significant improvement, similarly in both PG and TG groups, in the Action Naming test. Notably, although we measured a marginally significant improvement also for the Object Naming, we obtained statistically significant results only for the former. It has been shown that processing of action verbs is impaired in neurodegenerative disorders, PD included^93,94^. Accordingly, PD deficit in action naming can be considered within an embodied cognition framework^93,95^, in which language processing implying motor contents, requires a parallel neural simulation in the motor system^96^. This observation falls into the exploitation hypothesis^97,98^, basically stating that several higher order cognitive processes are grounded in the sensory-motor system. Following this hypothesis, linguistic deficits related to action verbs might result from damages at the level of the motor system^99,100^. We are among the first showing that after a complementary intervention of physical activity, PD patients improve performance in action naming test. Given the concomitant enhancement in motor performance, this result would be compatible with the embodied cognition and exploitation hypothesis or can be at least interpreted in light of those theoretical frameworks. Regardless of the hint to a specific theory, deficit in action naming in PD has been enumerated within the symptomatology of the early stages of the disease PD^94,101,102^. Showing an improvement in this domain is thus pivotal at clinical level and it advocates for a critical role of complementary interventions based on physical activity in PD neurorehabilitation.

We reported a significant improvement in the Ekman 60-Faces test^103^, specifically in the TG group. This neuropsychological test assesses emotion recognition from facial expressions. Although our PD sample did not show a critical impairment in this test at baseline, the PD patients performing tango during the intervention significantly improved their performance at the post evaluation session. Deficits in social cognition are nowadays recognised between the complex and extended cognitive deterioration affecting PD patients^104,105^. Accordingly, deficits in cognitive and affective theory of mind and in emotion recognition have been proven in PD populations since the early stages of the disease^106–109^. Furthermore, recent reviews suggest that deficits in emotion recognition in PD are particularly evident in the recognition of negative emotions^107^. A growing number of studies have been focusing on this topic in the last few years, since social cognition is still an under-investigated topic in clinical practice^110^. Within negative emotions, fear, anger and then sadness are the most difficult emotion to recognise in PD^111^. In the present study, we measured an improvement in global facial emotion recognition and a further significant improvement in the subscale related to sadness, in PD patients involved in the tango intervention. The specificity of the improvement in the TG suggests that elements other than the mere group-based format and physical activity in general concurred to this result. In this sense, we can only speculate that physical activity based on tango dance can promote more intense social interactions and a more sustained enjoyable context for patients with PD. In addition, during tango, face-to-face interaction and a particular attention to the intentions and emotional state of the partner are constantly present during the activity, which could probably have beneficial effects in the observation, discrimination and recognition of facial expressions. A recent systematic review and meta-analysis suggests that facial emotion recognition may be improved in neurodegenerative diseases with different methods ranging from cognitive, neurostimulation, to pharmacological approaches^112,113^. Within the cognitive approaches, authors proposed different tools including the Training of affect recognition^114,115^, originally developed for individuals with schizophrenia; the Gaia Training program^116^, error-less learning^117,118^, and the micro expression training tool^119,120^. In PD, it has been shown that facial emotion recognition improves under levodopa treatment^121^ and with a combination of pharmacological treatment and neurostimulation^122^. Comparably, other studies demonstrated an absence of performance improvement in emotion recognition after different treatments (for a review see Mirzai et al.^112^). To the best of our knowledge, we are among the first showing a significant improvement in facial emotion recognition after tango dance intervention in PD. This “collateral” finding highlights the potential of such an intervention in affecting PD pathology at different levels, even though not directly tackled by the activity itself.

We could then state that complementary intervention in PD based on dance, and particularly on tango dance, could be an effective strategy to limit PD pathology in the—often underestimated—impairment in facial emotion recognition. We gave just a first hint in this direction, but further detailed and tailored investigations are needed.

Capturing inter-individual differences and assessing the real efficacy of intervention protocols is an essential, albeit usually underestimated, goal of clinical studies. In this sense, a growing number of studies falling under the larger and broader umbrella of the so-called personalized precision medicine is accumulating^123^. In the present study, we started to fill this gap by implementing SD_R_ method to explore inter-individual differences in response to complementary intervention in PD. This method^65^ usually grounds on the comparison between an intervention and a control group, suggesting that the efficacy of an intervention protocol can be estimated only with respect to a comparison (no intervention) reference. The main rationale behind this—in its more simplified version—states that if the standard deviation of the changes in performance (in a specific variable of interest) of the intervention group is higher than that of the control group, true inter-individual differences of responses could be confirmed. Otherwise, group differences could be accounted for individual variation in trial-to-trial variability, natural physiological or psychological changes of the measured outcomes over time.

Here, we adapted this original proposal, using one intervention group as the control for the other. Although with the main group analyses, we did not get significant group differences (except for Mini-BESTtest and Ekman 60-Faces test), we explored true inter-individual differences in all the considered variables of interest.

For all the measures in which there was a significant main effect of Session, which suggested an improvement after the period of the intervention, we did not find any significant SD_R_. In our view, this result implies a comparable beneficial effect of tango and physiotherapy, with no true inter-individual differences in response to the different treatments. Accordingly, we could state, for example, that both interventions lead to a comparable decrease in motor sign severity (UPDRS-III), or comparable improvement in static and dynamic balance, at single individual level (e.g., Berg Balance Scale, TUG). At a clinical level, this result would suggest that the implementation of either tango or physiotherapy intervention, with no indications for one over the other, could lead to behavioural improvement in those variables.

On the other side, we found true inter-individual differences in several measures for which we did not report any significant difference between pre and post evaluation sessions in the main analysis (6 min walking test—distance, 10 m walking test, TMT A, TMT B, TMT B-A, Stroop test—time and the PAS total score). Specifically, we measured a significantly higher SD in the PG compared to the TG. In our view, this result suggests that physiotherapy intervention triggered a more variable change in performance in tests globally pertaining to gait, executive functions and anxiety. In other words, even though we did not find significant changes after the intervention with a standard parametric approach (2 × 2 Mixed Repeated measures ANOVA), the data on true inter-individual differences suggest that PG presented higher variability in performance change, which could indicate a more incisive end efficacy effect of this intervention in promoting behavioural modifications in PD patients.

We explored the issue of individual differences in response to treatment at a first, still general, level through the SD_R_ method, as already shown, for instance, in Alzheimer’s Disease^124^. We highlighted some useful observations indicating that TG and PG are comparably effective in promoting changes mainly at motor level, whereas group-based physiotherapy can promote further changes in performance in motor, cognitive and affective domains. However, we presented just a first attempt in the direction of precision medicine for complementary interventions in PD, the topic deserves further consideration and detailed analyses in future studies.

## Conclusions

Our findings support the implementation of physical activity as an effective complementary intervention in PD. In the present study, we adopted two types of intervention: group-based physiotherapy and Argentine tango dance. Our crucial findings demonstrated that both activities lead to behavioural improvements, mainly in the motor domain, which is critical in PD pathology. This improvement in the motor domain has an effect on a specific aspect of language, namely action naming, which was significantly improved by both interventions. Some differences emerged between the two intervention protocols. While physiotherapy seems to boost more variable changes in performance at individual level in measures pertaining to walking, executive functions and anxiety, tango led to significant improvement in facial emotion recognition, which is usually considered a second-order issue in PD pathology.

We acknowledge that the results and the following implications of the present study might be influenced by several limitations. The number of participants in each group is limited, which preclude generalisation of the findings. Furthermore, within the small sample size, patients exhibited highly variable levels of impairment (H&Y range), which again might have impacted our results. The limited beneficial effects we reported in the two groups, and the difference between them, might be probably linked to the short length of our interventions (4 months, see^79,80^ for longer protocols). It is known that plastic changes in the brain are experience-dependent^125,126^, and related to both the specific activity implemented and the state of the learning brain (health/pathological). Accordingly, the effectiveness of behavioural intervention in PD must be tested taking into consideration a longer period of active training. This will allow to capture more stable modifications at brain level, which will consequently reflected at behavioural level.

Nonetheless, the present investigation highlights overall the pivotal role that physical exercises—such as group-physiotherapy and Argentine tango dance—have in the preservation, stabilisation and in the slowdown of disease’s progression in neurodegenerative disorders, such as Parkinson’s Disease. The present findings thus points to the implementation of rehabilitation programs strongly founded on complementary intervention of physical activity provided at group level.

## Methods

### Participants

Twenty-four PD people were recruited for the present pilot study. Inclusion criteria were as follow: have a neurological diagnosis of idiopathic PD (according to the United Kingdom Parkinson’s Disease Society brain bank criteria^127^; no other neurological or medical problems; no evidence of structural abnormalities as proved by brain imaging; no deep brain stimulation (DBS); no particular injuries affecting movements or not having an excessive motor impairment preventing the participation to the project. Participants age ranged from 51 to 82 years old, and they were between Hoehn & Yahr stages II-III, all non-demented (see Table 1 for clinical feature of the sample). Participants were enrolled at the Centre for Neurocognitive Rehabilitation (CeRiN) of the Centre for Mind/Brain Sciences (CIMeC, University of Trento).

The study was approved by the local Ethical Committee at the University of Trento and it was in line with the Declaration of Helsinki (1964; amended in 2013, World Medical Association, 2013^128^). Participants read and signed an informed consent before participation.

### Procedure

This longitudinal project included three main phases: (1) Assessment (PRE); (2) Intervention; (3) Assessment (POST). In the assessment phases, all participants underwent: (1) a baseline clinical evaluation performed by licensed/certified neurologists and neuropsychologists; (2) a motor evaluation performed by a licensed/certified physiotherapist; (3) a neuroimaging evaluation, if the participant was compatible with magnetic resonance (MR) assessment. We did not consider the neuroimaging evaluation in the present study. All participants were tested while in their medication-ON condition.

Baseline evaluation (PRE) was conducted within two months before the start of the intervention phase, whereas post evaluation (POST) was conducted within a month after the end of the intervention on average.

Participants were assigned to two experimental groups enrolled in two distinct types of intervention: Tango and Physiotherapy. Group assignment was pseudo-random in order to have two groups (12 patients each) comparable in terms of age and sex (see Table 1).

The intervention, for both groups, comprised training sessions lasting about 60 min, two times a week, for a period of 4 months (32 sessions in total, from February to the end of May 2023).

Overall, the physical activity for both groups was progressive in nature, with an incremental and graded level of difficulty of the proposed exercises during the four months.

We explicitly asked participants not to follow other structured interventions while participating in our study protocol. However, we cannot completely exclude individual variability in the amount daily physical activity (such as walking, working or others) carried out by the patients.

### Interventions

#### Tango

In each session, the entire experimental group (12 patients) was present. The instructors were two tango instructors, with previous experience in developing courses of tango for people with PD.

During each training session, each patient was paired with a healthy volunteer, who was generally not expert in tango dance. Between the partners-pool, we excluded caregivers to avoid any family-related inconveniences/conflicts and ensure maximum social interaction with unknown people of the group. The patients-volunteer dancing couples were not fixed, but continuously changed during each session and across the entire intervention period. This exposure to dancing partner variety is common learning practice as it contributes to the development of dancing skills and sensibility. Instructions were tailored to the level of the group, with a high degree of flexibility to allow participants to slightly adapt the most difficult movements, if it was necessary.

The activity (Argentine Tango), along the whole intervention period, was divided in four main parts (cycles), principally aimed at maintaining, promoting and developing motor abilities. To this end, the instructors proposed exercises focused on improving posture and balance, improving walking and changing of walking direction, maintaining and reinforcing the strength of inferior limbs, further improving coordination (see Table S1 for a complete description of the program of Argentine Tango for PD). The course was conceived and programmed specifically for patients with PD, so the tango steps were adapted taking into account PD motor impairments.

Each session started with an individual welcome from the instructors and the assignment of volunteer-patient couples. The following preparatory phase consisted in a series of exercises mainly performed in static position. In this phase, the volunteers had principally a role of physical support and safety for the patients. In the central phase of the session, tango-specific exercises were carried out individually or in couples. Instructor showed the exercises followed by a first attempt from the learners. Subsequently, exercises were performed with specific music of argentine tango culture, as selected by the instructors. In the final phase, different exercises and music were proposed in order to dive into the communicative and socializing characteristics intrinsic to Argentine Tango.

Although the man usually has the leading role in tango, participant in this activity danced in both the leading and the following role, to ensure each participant moved forward and backward during dancing. The approach adopted allowed to implement and develop both self-directed and self-generated movements (leading role), and guided movements prompted by external cues from the partner (following role)^56^.

### Physiotherapy

Patients were divided in two subgroups, performing the intervention in two distinct but subsequent time during the same day. Accordingly, in each session two licensed physiotherapists followed six patients, thus ensuring the highest possible quality of training.

In each session, four areas were trained: (1)* Mobility exercises:* exercises were intended to train the different joints (e.g., cervical spine, dorso-lumbar spine), through exercise of inclination, rotation, flexion–extension. (2)* Balance exercises:* exercises included static in monopodalic, monopodalic with feedback and feedforward stimuli (e.g., grabbing a ball that was thrown at the person and throwing it back, maintaining the position); bipodalic exercises on unstable surfaces (soft mats), imbalance, and dynamic balance exercises. (3) *Muscle strengthening exercises:* these exercises included some main movements: squats/chair lifts/lunges to strengthen the glutes and quadriceps, exercises to strengthen arms and abdominal muscles. The exercises were performed using elastic bands with different resistances, dumbbells (1 or 2 kg) and fitballs. (4) *Exercises for cardiovascular health:* the last part of the session includes a circuit of three aerobic exercises: cyclette, step, forward and backward walking, with the addition of a motor coordination component. Every 5 min the patients changed station of the circuit, for a total of 15 min of cardiovascular exercise.

Each week, the difficulty of the exercises increased by changing different variables, such as resistance of the bands, weights of the dumbbells, height of the steps, numbers of repetitions and support during balance exercises. For a more detailed description of the procedure, refer to Table S2.

### Longitudinal assessments

PRE and POST evaluation sessions were identical, and they were conducted before and after the intervention phase, respectively. For the present study, we considered data pertaining to the motor, neurological and neuropsychological evaluations.

### Motor evaluation

A licensed physiotherapist individually conducted the motor assessment. The assessment comprised a battery of several practical tests and distinct questionnaires. Tests used to assess motor performance investigated *static balance* (mini-BESTest (miniBEST), Berg Balance Scale (BBS)); *dynamic balance* (Four Step Square (FSS), Timed Up and Go test (TUG) and 10-m walking test—fast condition (10mFast); *lower limb mobility and walking* (6-min walking test (6MWT), 30 s sit-to-stand (30STS), 5 time sit-to-stand (5STS), 10-m walking test—slow condition (10mSlow)) and *upper limb mobility* (9-hole peg test, 9hpt). We further included the following questionnaires: Fatigue Severity Scale (FaSS), Freezing of Gate Test (FGT), Activity-Specific Balance Confidence Scale (ABC) and Falls Efficacy Scale International (FES). For a detailed description of the motor tests used in the present protocol, refer to Supplementary Information, Methods section.

As a global measure of motor impairment, we used the motor subsection of the Movement Disorders Society Unified Parkinson disease Rating Scale (MDS-UPDRS-III,^129^). A neurologist performed the assessment for this scale.

### Neuropsychological evaluation

Participants underwent a battery of neuropsychological tests assessing different large-scale domains. In the battery of tests, we also included questionnaires on patients’ quality of life and specific inventories for the caregivers. According to the aim of the present study, we selected a subsample of the tests included in the neuropsychological battery. Regarding the cognitive domain, we selected measures of global cognitive status, visuo-spatial abilities, attention, executive functions, language and emotion recognition. A licensed neuropsychologist delivered the assessment.

#### Cognitive domain

We used the Montreal Cognitive Assessment (MoCA) as an index of *global cognitive status*^130^. *Visuo-spatial abilities* were tested with the Judgement of Line Orientation test and the Facial Recognition test^131^; Attentive Matrices^132^ and Trial Making Test (TMT; part A and B; Siciliano et al.^133^; Giovagnoli et al.^134^) were used as measures of *attention*, selective and divided, respectively. *Executive functions* were assessed with the TMT (part B-A; Siciliano et al.^133^; Giovagnoli et al.^134^), the Stroop task^135^ and the Phonemic Verbal Fluency test^136^. We also included two tests for *language*: the Semantic Fluency test^137^ and the Picture Naming of objects and actions^138,139^. We used the Ekman 60 Faces [117] to test facial emotion recognition.

#### Affective domain

Mood changes were evaluated in relation to symptoms of depression, anxiety and apathy, through the Geriatric Depression Scale (GDS)^140^, the Parkinson Anxiety Scale (PAS)^141^ and the Apathy Evaluation scale (AES)^142^, respectively.

Moreover, we delivered the Parkinson’s Disease Questionnaire-39 (PDQ-39)^143^ to explore potential effects of intervention on perceived quality of life.

### Statistical analysis

We performed preliminary analyses to test data distribution (Shapiro–Wilk test) and to evaluate group differences in demographic variables (i.e., age, sex, education, H&Y, disease duration and UPDRS-III), through two-sample independent Student t-test (for normally distributed data) or Mann–Whitney test (for not-normally distributed data).

To test the effectiveness of the intervention and to highlight potential differences between the two proposed physical activities, we used mixed 2 × 2 Repeated Measures ANOVA with *Group* (TG, PG) as between subject factor, and *Session* (PRE, POST) as within subject factor.

We used separate ANOVA for each variable of interest, further exploring the results with post-hoc tests (t-test). We used the adjusted scores according to Italian normative values for each neuropsychological variable. As a control analysis, to assess the strength of the null hypothesis, we computed also the Bayes Factor^144^ (BF_10_). We added this information in the analyses in which we reported significant results.

While the group-level analysis gives crucial information on the global effect of the interventions proposed, we cannot underestimate the potential inter-individual differences occurred in response to the proposed activities. Accordingly, we used the concept of *“true inter-individual differences”* explained by Atkinson and Batterham^65^ to explore this issue.

True inter-individual differences in intervention outcomes were calculated, for each variable of interest, as the difference between the standard deviation (SD) of change of the two groups. The resulting SD represents the “true inter-individual variation in response to intervention, adjusted for the influence of random biological variation measurement error (removal of “noise”)” (Atkinson^65^). Usually, it is computed based on the data distribution of an intervention group and a control group, and it is conceived as representing the “true” efficacy of the intervention protocol. In our case, we had two intervention groups, therefore we used the concept of “*true inter-individual differences*” with a slightly different approach. We calculated this measure as follow:

$$SDR = \sqrt {SDT^{2} - SDP^{2} }$$

where SD_R_ is the SD of the true individual difference in response to the intervention (tango as it is in the formula); SD_T_ e SD_P_ are the SDs of the change in performance (pre-post scores) for tango (SD_T_) and physiotherapy (SD_P_) group, respectively. Note that we arbitrarily used the tango group as the first factor in the formula. However, SD of change in either the tango group or the physiotherapy group could be greater than the other. Therefore, for each specific variable of interest, in case the SD of change was higher in the tango group, the SD_R_ had positive values; otherwise, in case SD of change was greater in the physiotherapy group, we switched the order of the factors within the formula in order to calculate a value, which retrospectively was converted in negative (*− 1). Accordingly, positive SD_R_ indicated higher SD of change in the tango group and negative SD_R_ indicated higher SD of change in the physiotherapy. Differently from the procedures used in previous publications using this method^65,124^, we statistically validated this measure through nonparametric permutation testing. To calculate a statistical value of significance (*p* < 0.05), we randomly shuffled the participants between the two groups (n° permutation = 10,000), calculating the SD_R_ value in each permutation, thus obtaining a distribution of permuted SD_R_. We calculated significance by counting the number of permuted SD_R_ higher/lower than the real SD_R_ and further dividing by the number of iterations.

We hypothesised two interpretations for the SD_R_ values. For the measures in which we reported a significant effect of improvement from PRE to POST session in the main analysis, a higher SD_R_ in one group gives indications of a more stable and consistent effect of improvement in the other group. Conversely, for the measures in which we did not reported any significant improvement in the main analysis, higher SD_R_ in one group can be interpreted as indication for true inter-individual difference in response to the intervention, suggesting a higher variability of the effect of the activity for that group in changing performance in the specific test.

### Supplementary Information

Supplementary Information.

## Data Availability

The data supporting the findings of this study are available on request from the corresponding author.
