# Supplementary material for: Tango and physiotherapy interventions in Parkinson’s disease: a pilot study on efficacy outcomes on motor and cognitive skills
Source: Sci Rep. 2024 May 24;14:11855. doi: 10.1038/s41598-024-62786-6 (PMC11126665; doi:10.1038/s41598-024-62786-6)
Supplement: Supplementary file 1 — Supplementary Information. [file 41598_2024_62786_MOESM1_ESM.docx]

**SUPPLEMENTARY INFORMATION**

**Tango and Physiotherapy interventions in Parkinson’s Disease:**

**A pilot study on efficacy outcomes on motor and cognitive skills**

**Methods**

**Interventions**

In this section, we report a complete description of the intervention we implemented in our protocol, using a validated instrument, the Template for Intervention Description and Replication – TIDieR - ^1^. Indications for downloading the Template can be found at <https://www.bmj.com/content/348/bmj.g1687>. This instrument is intended to give necessary information for the replicability of the intervention developed.

**Table S1** | **ARGENTINE TANGO INTERVENTION**

| **Brief Name** | |
| --- | --- |
| 1. | Tango Intervention / Tango: a complementary therapy for Parkinson’s Disease |
| **Why** | |
| 2. | Tango dance aimed mainly to achieve the general objectives, shared with the physical and motor therapies, of maintaining and promoting the general physical health of the patients. The focus was both on those aspects usually impaired in Parkinson’s disease, such as balance skills, muscle strength and coordination, but also to the more “social” aspect of the intervention, related to social interaction and entertainment |
| **What** | |
| 3. | Materials: Tango classes did not require any specific instrument or material for participants, who were allowed to get dressed as comfortable as they prefer. During the program, teachers selected different music tracks from the vast Argentine Tango culture |
| 4. | Procedures: individual sessions were structured as follow:   1. Hospitality: with a background of music, teachers formed the couples taking into account the planned topic, personal abilities and personal problems 2. Preparatory Exercises: a series of exercises aimed at improving posture, balance and facilitating directional changes while walking. The exercises were performed while sitting or standing, rarely while moving 3. Specific Tango Exercises: exercises could be individual or performed in couples. First teachers explained and demonstrated the dance steps /movements of the individual session. The participants performed the movements without and finally with music background. During the exercise the teachers constantly monitored the difficulties of the patients, intervening when necessary. High attention was paid to the level of fatigue of the group. Music was carefully selected by searching for melodies and rhythm suited to the proposed technical bases 4. Conclusion: in the last part, teachers proposed music and exercises that included the communicative, playful and socializing characteristics intrinsic to Argentine Tango   The entire program was divided in four main cycles:   - Cycle 1: *Main Objectives*. Perception of one’s own balance and posture; recognition of the support and dealing with body axis and weight; coordination of movement with breathing; sharing movement in the couple; space management; learning new motor patterns   *Technical objectives of Tango*: connection with the partner; movement intention and preparation; starting, stopping and direction; walk forwards and backwards, technical hug; base 6; roundabout   - Cycle 2: *Main Objectives*. Perception of direction and length of one’s step; use and management of lower limb strength; creation of new motor schemas; interpretative creativity   *Technical objectives of Tango*: contrast in the connection with the partner; side steps; the length of the steps in the musical variation; tango hug; base 8 with crossover   - Cycle 3: *Main Objectives*. Torso mobility and shoulder joints; recognize and manage stability with the partner; elicit and react to different stimuli; learning new motor schemas   *Technical objectives of Tango*: dissociation; pivot; ocho adelante (eight forward); ocho atràs (eight backward); sharing dynamic balance with the partner   - Cycle 4: *Main Objectives*. Changes in direction; overcoming obstacles; Time and space evaluation; execution of a complex sequence.   *Technical objectives of Tango*: Crusado; parade; mordida; scavalco; cunida; giro (turn around) |
| **Who provided** | |
| 5. | Patients were supervised by 2 teachers (one male, one female) with a long-standing experience in Argentine Tango classes and with specific experience with Parkinson’s Disease patients. The teachers already delivered classes of Tango for PD in their career. Patients were supported by a group of volunteers (partner), who were mainly not familiar with Argentine Tango. Partners were selected intentionally as coming outside patient’s family, in order to promote social interaction with unknown people and avoid personal-related issues. During the program, the couples were always constituted by a patient and a volunteer (partner) |
| **How** | |
| 6. | Tango dance classes were delivered in presence, and as generally is conducted as a group activity. Both teachers were always present, volunteers were randomly assigned to patients in each session. The whole group (more or less 24 individuals) performed the activity together. If the number of volunteers exceeded the number of patients, couples were formed between volunteers |
| **Where** | |
| 7. | The activity was carried out in an indoor structure (around 60 meter squared), with a wood floor, usually dedicated to dance activity. The site was located in the city of Rovereto (TN, Italy) |
| **When and How Much** | |
| 8. | Each training session lasted 1 hour, twice a week, from February 2023 to May 2023, for a total of 32 sessions. The entire program was divided in four cycles of eight hours each. Each week was characterized by the maintenance of the preceding steps plus additional exercises with an incremental level of difficulty. |
| **Tailoring** | |
| 9. | The activity was planned to be standardized. However, the teachers, in some cases, adjusted and adapted the exercises to the capacity of the single patient. For example, the intensity and the duration of the exercise were reduced when necessary |
| **Modifications** | |
| 10. | The intervention was generally delivered as programmed in advance. Some little changes have been done, but mainly to overcome patients difficulties in performing specific steps or exercises |
| **How well** | |
| 11. | Patient’s motivation in participating was constantly and intrinsically high. Nonetheless, teachers kept the patients motivated by changing weekly the type of exercise delivered and by promoting sharing of ideas and comments, by promoting in general social interaction and finally sharing the passion for Argentine Tango dance, music and culture. |
| 12. | The intervention adherence was assessed by calculating the frequency rate of participation. The mean frequency rate was about 92%, without drop-outs |

**Table S2** | **PHYSIOTHERAPY INTERVENTION**

| **Brief Name** | |
| --- | --- |
| 1. | Physiotherapy Intervention /Motor Training |
| **Why** | |
| 2. | The physical activity was structured to improve the general physical health of the patient, with a particular focus on improving those aspects that are mostly impaired by the Parkinson’s disease such as balance skills, muscle strength and aerobic resistance |
| **What** | |
| 3. | Materials: During the training, a set of different materials was required to perform the exercises and to allow the therapists to change the modality of the training over the time. The material consisted of balls, fit balls, step platforms with adjustable height, elastic resistance bands, tennis balls, yoga/soft mats and cycle ergometers with adjustable resistance. |
| 4. | Procedures: the group sessions were divided into 4 main parts, of 15 minutes each, that consisted of a series of exercises performed in the same order for each group:   - Mobility training   The exercises focused mainly on improving the mobility of the joints of the cervical spine (flexion, extension and lateral flexion), thoracic-lumbar spine (rotation and lateral flexion), hip joints (flexion and extension) and ankle joints.   - Balance training   For what concerns the balance training, the exercises performed varied between static monopodalic exercises, monopodalic exercises with feedback and feedforward stimuli (catching a ball and throwing it back, maintaining balance in monopodalic position), bipodalic exercises on unstable surfaces (soft mats) and lateral imbalance, and dynamic balance exercises (during walking).   - Muscle strength training   The exercises for the muscle strengthening were modified each week, keeping the focus on some specific movements: squat/sit-to-stand/lunges for glutes and quadriceps strengthening, exercises for the strengthening of the anterior and posterior muscle chains of the upper limbs and exercises for abs and core strengthening. The activity was performed using fit balls, dumbbells (1 or 2 kg) and elastic resistance bands   - Cardiovascular endurance training   The last part of the session was dedicated to aerobic and resistance exercises. The activity consisted of a circuit training composed by a set of 3 exercises: cycle ergometer, step ups and walking combined with some coordination exercises. The patients changed the exercise every 5 minutes in order to complete this part in 15 minutes |
| **Who provided** | |
| 5. | Each group of patients was supervised by 2 professional physiotherapists with 3-4 years of experience in neurological and orthopedic physical rehabilitation |
| **How** | |
| 6. | The training was a standardized, in-presence physical activity. The 12 patients were divided into 2 groups of 6 and they performed the same exercises in the same order |
| **Where** | |
| 7. | The activity was carried out in an indoor structure (around 30 meter squared), dedicated to the physical activity. The site was located in the city of Rovereto (TN, Italy) |
| **When and How Much** | |
| 8. | Each training session lasted 1 hour, twice a week, from February 2023 to May 2023, for a total of 32 sessions. Each week the intensity of the exercises and the number of repetitions was increased |
| **Tailoring** | |
| 9. | The activity was planned to be standardized. However, in some cases the intensity and the number of repetitions were adjusted and adapted to the capacity of the single patient |
| **Modifications** | |
| 10. | At the beginning of the training some modifications were already planned. We progressively increased the number of repetitions, the weight of the dumbbells and the resistance of the elastic bands. However, during the training we had to modify and personalize some exercises for one patient that had significant difficulties due to frequent freezing episodes |
| **How well** | |
| 11. | We tried to keep the patients motivated by changing weekly the type of exercise delivered and by settings exercises that allowed them to interact with each other, so that they could also socialize and making the training more entertaining |
| 12. | The intervention adherence was assessed by calculating the frequency rate of participation. The mean frequency rate was about 80%, with 2 drop-outs |

**Motor assessment**

Motor assessment was performed by a professional physiotherapist and was aimed to evaluate different aspects of motor abilities in PD patients. We used a series of practical exercises and different questionnaires. Here a complete description.

- **Six-minutes walking test.** It evaluates the distance covered over a time of 6 minutes and it is used to assess aerobic capacity and endurance. Before, after and during the test some parameters are monitored such as heart rate, oxygen saturation, blood pressure, perception of effort and number of steps. It belongs to the “activity” category of the International Classification of Functioning, Disabilities and Health (ICF) and it takes about 10 minutes to be completed ^2^.
- **mini-BESTest**. This is a reduced version of BESTtest that evaluates balance skills. The evaluated areas are: anticipatory postural control, reactive postural control, sensory orientation and dynamic gait. It belongs to the “structure and activity” category of the ICF classification and it takes about 15-20 minutes to be completed ^3^.
- **Berg Balance Scale.** It is used to determine a patient's ability (or inability) to safely balance during a series of predetermined tasks. The physiotherapist rates the patient’s performance on a 14 item list, with each item consisting of a five-point ordinal scale ranging from 0 to 4, with 0 indicating the lowest level of function and 4 the highest level of function. It belongs to the “activity” category of the ICF classification and it takes about 20 minutes to be completed ^4^.
- **The 10 Meter Walk Test.** It is used to assess walking speed over a distance of 10 meters during a comfortable speed and a fast paced walking. It belongs to the “structure and activity” category of the ICF classification and it less than 5 minutes to be completed ^5^.
- **30 seconds sit to stand and 5 time sit to stand**. It measures the strength and endurance of lower limb in older adults. This test has been developed to overcome the “floor effect” of the “5 repetition sit to stand” test and “10 repetition sit to stand” test. It belongs to the “structure and activity” category of the ICF classification and it takes less than 5 minutes to complete ^6^.
- **Four Square Step.** It is used to assess dynamic stability and coordination. It determines the ability of the subject to step over low objects forward, sideways and backward.

It belongs to the “activity” category of the ICF classification and it takes less than 5 minutes to be completed ^7^.

- **Timed Up and Go.** It is used to determine fall risk and measure the progress of balance, sit to stand and walking. It belongs to the “activity” category of the ICF classification and it takes about 1-2 minutes to be completed ^8^.
- **9 Hole Peg Test.** The Nine-Hole Peg Test (9HPT) is used to measure finger dexterity during a functional task. It belongs to the “activity” category of the ICF classification and it takes less than 5 minutes to be completed ^9^.
- **Fatigue Severity scale.** It’s 9-item scale which measures the severity of fatigue and its effect on a person's activities and lifestyle in patients with a variety of disorders. It belongs to the “structure” category of the ICF classification and it takes less than 5 minutes to be completed ^10^.
- **Freezing of Gate.** It’s a self-reported questionnaire that measures the entity of the freezing episodes related to the gait and other motor aspect (starting and turning ability). It belongs to the “activity” category of the ICF classification and it takes less than 5 minutes to be completed ^11^.
- **Activity specific balance confidence scale.** Self-reported questionnaire that measures an individual’s confidence during ambulatory activities without falling or experiencing a sense of unsteadiness. It belongs to the “activity” category of the ICF classification and takes about 5-10 minutes to be completed ^12^.
- **Falls efficacy scale international.** Self-reported questionnaire that measures the fear of falling in different daily activities. It belongs to the “activity” category of the ICF classification and takes about 5-10 minutes to be completed ^13^.

**Table S3** | **BASELINE between-groups comparison: MOTOR MEASURES**

|  | **TG (n=12)** | | **PG (n=12)** | **Statistics** | |
| --- | --- | --- | --- | --- | --- |
| **MOTOR MEASURES** | | | | | |
| ***Static Balance*** | | | | | |
| ***Mini BESTest (miniBest)*** | | 26.33 [7.29] | 23.83 [5.11] | | U=104.5, p=0.06, r=0.45 |
| ***Berg Balance Scale (BBS)*** | | 52.08 [5.88] | 50.83 [4.88] | | U=95.00, p=0.19, r=0.32 |
| ***Dynamic Balance*** | | | | | |
| ***Four Squares Step (FSS)*** | | Best: 10.36 [2.18]  Mean: 10.91 [2.31] | 10.02 [2.48]  10.990 [2.04] | | t =0.45, p=0.66, Cohen’s d =0.18  U=67, p=0.80, r =0.07 |
| ***Timed Up and Go (TUG)*** | | 9.55 [2.71] | 9.47 [1.87] | | U=61.5, p=0.56, r =0.15 |
| ***Ten meters walking, fast condition (10mFast)*** | | Mean: 3.69 [0.60]  Speed: 2.78 [0.48] | 7.45 [12.07]  2.39 [0.82] | | U=53.00, p=0.29, r =0.26  U=91.00, p=0.29, r =0.03 |
| ***Lower-Limb mobility and walking*** | | | | | |
| ***6-meters walking test - Distance (6MWT)*** | | 370.88 [46.12] | 343.32 [105.95] | | U=87.00, p=0.40, r =0.21 |
| ***30 second Seat-to-Stand (30STS)*** | | Best: 14.50 [3.66] Mean: 13.95 [3.63] | 15.17 [3.90]  14.5 [3.83] | | U=76.50, p=0.81, r =0.06  U=78.50, p=0.73, r =0.09 |
| ***5 time Seat-to-Stand (5STS)*** | | Best: 12.53 [6.55]  Mean: 13.24 [6.46] | 10.98 [2.03]  11.89 [1.94] | | U=71.00, p=0.98, r =0.01  U=67.00, p=0.78, r =0.07 |
| ***Ten meters walking, slow condition (10mSlow)*** | | Mean: 4.76 [0.51]  Speed: 2.12 [0.23] | 6.51 [0.44]  1.92 [0.56] | | U=49.00, p=0.19, r =0.32  U=95.00, p=0.20, r =0.32 |
| ***Upper-Limb Mobility*** | | | | | |
| ***9-Hole Peg Test (9hpt)***  ***-right*** | | 30.89 [7.75] | 32.03 [10.59] | | t=0.30, p=0.77, Cohen’s d=0.12 |
| ***9-Hole Peg Test (9hpt)***  ***-left*** | | 31.58 [5.33] | 34.07 [9.89] | | t=0.77, p=0.45, Cohen’s d=0.32 |
| ***Questionnaires*** | | | | | |
| ***Fatigue Severity Scale (FaSS)*** | | 37.50 [13.96] | 34.25 [16.48] | | U=81.50, p=0.60, r =0.13 |
| ***Freezing of Gate Test (FGT)*** | | 2.58 [6.07] | 3.25 [8.24] | | U=72.00, p=1.00, r =0.00 |
| ***Activity-Specific Balance Confidence Scale (ABC)*** | | 78.54 [20.62] | 83.91 [18.36] | | U=66.00, p=0.75, r =0.08 |
| ***Falls Efficacy Scale International (FES)*** | | 26.17 [10.77] | 23.67 [8.53] | | U=85.50, p=0.45, r =0.19 |

**Table S4. |** **BASELINE between-groups comparison: Cognitive Measures**

|  | **TG (n=12)** | | **PG (n=12)** | **Statistics** | |
| --- | --- | --- | --- | --- | --- |
| **COGNITIVE MEASURES** | | | | | |
| ***Global Cognitive Status*** | | | | | |
| ***Montreal Cognitive Assessment (MoCA)*** | | 23.10 [3.57] | 22.15 [2.52] | | t=0.75, p=0.46, Cohen’s d =0.31 |
| ***Verbal and Visuo-spatial short-Term Memory*** | | | | | |
| ***Digit Span – forward (Digit-F)*** | | 6.09 [1.15] | 5.62 [1.02] | | t =1.05, p=0.30, Cohen’s d =0.43 |
| ***Working Memory*** | | | | | |
| ***Digit Span – backword (Digit-B)*** | | 4.33 [0.78] | 4.05 [0.51] | | t =1.02, p=0.32, Cohen’s d =0.42 |
| ***Corsi Test (Corsi)*** | | 5.07 [0.75] | 4.80 [0.66] | | U=87.00, p=0.40, r=0.21 |
| ***Learning and Long-Term Memory*** | | | | | |
| ***RAVLT*** | | Index of learning ability: 45.46 [13.82]  Verbal long–term memory: 9.24 [4.56] | 44.06 [13.52]  9.54 [4.09] | | t =0.25, p=0.80, Cohen’s d =0.10  t =0.17, p=0.87, Cohen’s d =0.07 |
| ***Rey-Osterrieth Complex Figure Test - Recall*** | | 17.56 [7.61] | 15.54 [5.56] | | t =0.74, p=0.47, Cohen’s d =0.30 |
| ***Visuo-spatial abilities and planning*** | | | | | |
| ***Benton Facial Recognition Test*** | | 45.25 [4.41] | 44.17 [5.20] | | t=0.55, p=0.59, Cohen’s d=0.22 |
| ***Rey-Osterrieth Complex Figure Test - Copy*** | | 31.81 [3.30] | 29.52 [5.60] | | U=98.50, p=0.13, r=0.37 |
| ***Attention*** | | | | | |
| ***Attentive Matrices*** | | 46.96 [6.83] | 45.69 [9.23] | | t=0.38, p=0.70, Cohen’s d=0.16 |
| ***Trial Making Test***  ***-part A-*** | | 30.67 [13.62] | 29.50 [18.05] | | U=79.00, p=0.71, r=0.10 |
| ***Trial Making Test***  ***-part B-*** | | 89.17 [73.55] | 186.5 [198.6] | | U=54.50, p=0.33, r=0.24 |
| ***Executive Functions*** | | | | | |
| ***Trial Making Test –***  ***Part B-A*** | | 57.58 [65.53] | 153.42 [177.55] | | U=51.50, p=0.25, r=0.28 |
| ***Stroop Test*** | | Errors: 0.44 [1.11]  Time: 15.35 [9.02] | 0.94 [3.41]  23.68 [15.21] | | U=81.50, p=0.49, r=0.13  U=50.00, p=0.21, r=0.31 |
| ***Phonemic Verbal Fluency test*** | | 43.58 [11.10] | 35.87 [15.39] | | t=0.86, p=0.40, Cohen’s d=0.35 |
| ***Language*** | | | | | |
| ***Semantic fluency Test*** | | 48.52 [13.06] | 44.18 [11.57] | | t=0.55, p=0.59, Cohen’s d=0.22 |
| ***Naming Figures*** | | 47.39 [0.98] | 46.67 [1.74] | | U=93.50, p=0.19, r =0.30 |
| ***Naming Verbs*** | | 48.37 [3.55] | 45.88 [4.68] | | U=95.5, p=0.18, r =0.33 |
| ***Emotion Recognition*** | | | | | |
| ***Ekman 60-Faces*** | | 49.92 [5.39] | 47.27 [6.00] | | t=1.09, p=0.29, Cohen’s d=0.46 |

**Table S5 |** **BASELINE between-groups comparison: Affective Measures and Perceived Quality of Life**

|  | **TG (n=12)** | **PG (n=12)** | **Statistics** |
| --- | --- | --- | --- |
| **AFFECTIVE** | | | |
| ***Parkinson Anxiety Scale – Global Score (PAS)*** | 9.83 [7.12] | 15.75 [5.83] | t=2.23, p=0.04,  Cohen’s d=0.91 |
| ***Geriatric Depression scale – Global Score*** | 8.25 [6.02] | 11.67 [6.96] | t=1.29, p=0.21,  Cohen’s d=0.53 |
| ***Apathy Evaluation Scale Global Score (AES)*** | 30.42 [8.14] | 32.42 [5.16] | t=0.72, p=0.48,  Cohen’s d=0.72 |
| **PERCEIVED QUALITY OF LIFE** | | | |
| ***PDQ39-Tot*** | 19.47 [13.26] | 18.81 [10.51] | t=0.14, p=0.89,  Cohen’s d=0.06 |
| ***PDQ39-Mobility*** | 14.79 [15.1] | 23.12 [19.75] | t= 1.16, p=0.25,  Cohen’s d= 0.47 |
| ***PDQ39-ADL*** | 20.83 [21.096] | 9.65 [13.61] | U=96.00, p=0.17,  r=0.33 |
| ***PDQ39-Emotional wellbeing*** | 20.83 [21.97] | 24.30 [11.76] | t=0.48, p=0.63,  Cohen’s d= 0.20 |
| ***PDQ39-Stigma*** | 17.71 [20.10] | 15.10 [12.35] | t=0.38, p=0.71,  Cohen’s d=0.16 |
| ***PDQ39-Social support*** | 6.24 [10.12] | 5.56 [11.42] | U=81.50, p=0.53,  r=0.13 |
| ***PDQ39-Cognition*** | 26.04 [19.91] | 21.35 [16.53] | t=0.63, p=0.54,  Cohen’s d=0.26 |
| ***PDQ39-Communication*** | 22.91 [21.94] | 25.00 [23.02] | t=0.23, p=0.82,  Cohen’s d=0.09 |
| ***PDQ39-Bodily discomfort*** | 26.40 [30.32] | 25.69 [20.24] | t=0.07, p=0.95,  Cohen’s d=0.03 |

**Table S6. |** **True individual differences in performance change**

| Behavioural Measure | SDr | Group 1 | p value (permutation) |
| --- | --- | --- | --- |
| **MOTOR** | | | |
| UPDRS III | -2.38 | Physiotherapy | 0.36 |
| 10 meters - slow (mean) | -3.73 | Physiotherapy | 0.17 |
| 10 meters - fast (mean) | -12.03 | Physiotherapy | <0.001* |
| Four Square Step (mean) | -0.85 | Physiotherapy | 0.47 |
| Timed Up & Go | -1.55 | Physiotherapy | 0.44 |
| miniBESTest | 1.82 | Tango | 0.43 |
| 5 time Sit-to-Stand | 4.80 | Tango | 0.06 |
| 30 seconds Sit-to-Stand | -1.55 | Physiotherapy | 0.43 |
| Berg Balance scale | -3.61 | Physiotherapy | 0.19 |
| 6 minutes walking (distance) | -47.56 | Physiotherapy | <0.001* |
| **COGNITIVE** | | | |
| MoCA | -2.54 | Physiotherapy | 0.35 |
| Digit Backward | -0.33 | Physiotherapy | 0.48 |
| TMT - A | -12.95 | Physiotherapy | <0.001* |
| TMT – B | -117.94 | Physiotherapy | <0.001* |
| TMT – B-A | -116.32 | Physiotherapy | <0.001* |
| Stroop errors | -2.44 | Physiotherapy | 0.36 |
| Stroop Time | -15.77 | Physiotherapy | <0.001* |
| Phonemic Fluency | 3.83 | Tango | 0.16 |
| Corsi | 0.50 | Tango | 0.51 |
| Attentive Matrices | 1.69 | Tango | 0.45 |
| Naming figures | -1.03 | Physiotherapy | 0.46 |
| Naming verbs | 2.054 | Tango | 0.41 |
| Ekman-60 | -2.49 | Physiotherapy | 0.35 |
| Unknown Face Recognition test | 3.03 | Tango | 0.27 |
| **AFFECTIVE** |  |  |  |
| Geriatric Depression Scale (GDS) | 2.81 | Tango | 0.30 |
| Parkinson Anxiety Scale (PAS) | -5.53 | Physiotherapy | 0.02* |
| Apathy Evaluation Scale (AES) | 2.23 | Tango | 2.23 |

**References**

1. Hoffmann, T. C. *et al.* Better Reporting of Interventions: Template for Intervention Description and Replication (TIDieR) Checklist and Guide. *Gesundheitswesen* **78**, 175–188 (2016).

2. Reesink, H. J. *et al.* ATS statement : Guidelines for the sixmin walk test. *Am J Respir Crit Care Med* **166**, 111–117 (2002).

3. Leddy, A. L., Crowner, B. E. & Earhart, G. M. Utility of the Mini-BESTest, BESTest, and BESTest Sections for Balance Assessments in Individuals with Parkinson Disease. *J. Neurol. Phys. Ther.* **35**, 90 (2011).

4. Berg, K. O., Wood-Dauphinee, S. L. Williams, J. I. & Maki, B. Measuring balance in the elderly: validation of an instrument. *Can. J. Public Heal.* **83**, S7–S11 (1992).

5. Peters, D. M., Fritz, S. L. & Krotish, D. E. Assessing the reliability and validity of a shorter walk test compared with the 10-Meter Walk Test for measurements of gait speed in healthy, older adults. *J. Geriatr. Phys. Ther.* **36**, 24–30 (2013).

6. McCarthy, E. K., Horvat, M. A., Holtsberg, P. A. & Wisenbaker, J. M. Repeated Chair Stands as a Measure of Lower Limb Strength in Sexagenarian Women. *Journals Gerontol. Ser. A* **59**, 1207–1212 (2004).

7. Dite, W. & Temple, V. A. A clinical test of stepping and change of direction to identify multiple falling older adults. *Arch. Phys. Med. Rehabil.* **83**, 1566–1571 (2002).

8. Beauchet, O. *et al.* Timed up and go test and risk of falls in older adults: A systematic review. *J. Nutr. Heal. Aging* **15**, 933–938 (2011).

9. Feys, P. *et al.* The Nine-Hole Peg Test as a manual dexterity performance measure for multiple sclerosis. *Mult. Scler.* **23**, 711–720 (2017).

10. Krupp, L. B., Larocca, N. G., Muir Nash, J. & Steinberg, A. D. The Fatigue Severity Scale: Application to Patients With Multiple Sclerosis and Systemic Lupus Erythematosus. *Arch. Neurol.* **46**, 1121–1123 (1989).

11. Giladi, N. *et al.* Validation of the freezing of gait questionnaire in patients with Parkinson’s disease. *Mov. Disord.* **24**, 655–661 (2009).

12. Powell, L. E. & Myers, A. M. The Activities-specific Balance Confidence (ABC) Scale. *Journals Gerontol. Ser. A* **50A**, M28–M34 (1995).

13. Tinetti, M. E., Richman, D. & Powell, L. Falls Efficacy as a Measure of Fear of Falling. *J. Gerontol.* **45**, P239–P243 (1990).
